# Anthropometric Analysis of Distal Femur Parameters in the Eastern Uttar-Pradesh Population

**DOI:** 10.7759/cureus.33945

**Published:** 2023-01-18

**Authors:** Amit K Nayak, Chetan Sahni, Mayank Gupta, Praveen K Tiwari, Anand Mishra, Deepa Devadas

**Affiliations:** 1 Anatomy, Institute of Medical Sciences, Banaras Hindu University, Varanasi, IND; 2 Forensic Medicine, Institute of Medical Sciences, Banaras Hindu University, Varanasi, IND

**Keywords:** lateral condyle femur, medial condyle femur, femur anthropometry, distal femur, femur

## Abstract

Introduction: Anthropometric measurements of the distal femoral fragment play a crucial role in prosthesis design during arthroplastic knee surgeries and offer valuable clues for stature estimation in forensic investigations. The present study is an attempt to assess various anthropometric parameters of the distal femur in this regard.

Materials and methods: A total of 96 intact dry femora were anthropometrically assessed using digital Vernier calipers. The femoral length was measured using an osteometric table. The torsion angle was calculated with an analog goniometer. The various parameters studied included: medial condyle length and thickness, lateral condyle length and thickness, bicondylar width, intercondylar width, intercondylar depth, torsional angle, and femoral length. The data obtained were statistically analyzed using SPSS software (IBM Corp., Armonk, NY, USA).

Results: Mean medial condyle length was 57.38±4.47mm and thickness was 24.53±2.27mm. Mean lateral condyle length and thickness were found to be 58.49±4.3mm and 25.33±3.15mm respectively. Mean bicondylar width was 71.96±6.73mm, mean intercondylar width 21.86±2.71mm, and the intercondylar depth 27.04±2.59mm respectively. The average femur length was 41.87±3.31mm and the average torsion angle was 20.19°±6.99°. Significant correlations were observed between distal femoral parameters. Lateral condyle length showed maximum correlation with other parameters. Femur length was found to correlate significantly with all parameters except medial condyle thickness. Torsion angle was significantly correlated with lateral condyle length and femur length only.

Discussion: The findings of this study show considerable variation from those of other studies done within India. This proves that distal femoral anthropometry has regional variations. These data can aid sports physicians and orthopedic surgeons with implant designing and forensic experts during investigations.

## Introduction

The femur is the bone of the thigh. It has a proximal end, a distal end, and an intervening shaft. The distal end presents a double condyle that articulates with the tibia to form the knee joint complex. Anteriorly the condyles are confluent, but posteriorly they are separated by a deep intercondylar fossa and project beyond the plane of the popliteal surface. The lateral wall of the intercondylar fossa is formed by the medial surface of the lateral condyle, whereas the medial wall of the intercondylar fossa is formed by the lateral surface of the medial condyle. The lateral condyle is larger anteroposteriorly than the medial condyle. The medial condyle has a bulging, convex medial aspect which is easily palpable [1].

Measurements of the distal femur are crucial for total knee arthroplasty and there exists a high correlation between distal femur morphometry, femur length and femur width [2,3]. There exists a moderate to high correlation between femur length and fragment measurements. Femur anthropometry is also crucial in forensic investigations as the stature of an individual can be calculated from a fragmented femur [4]. Various regression formulae, specific to different populations, have been formed to calculate individual stature [5]. Various radiological studies have also been done to measure various parameters of the femur in living, but values measured by digital X-ray are slightly lesser than those measured from dry bone [6].

The present study has been planned to obtain the values of different parameters of the distal femur that will be helpful to orthopedic surgeons, forensic experts and sports physicians in their respective fields.

## Materials and methods

**Figure 1:**
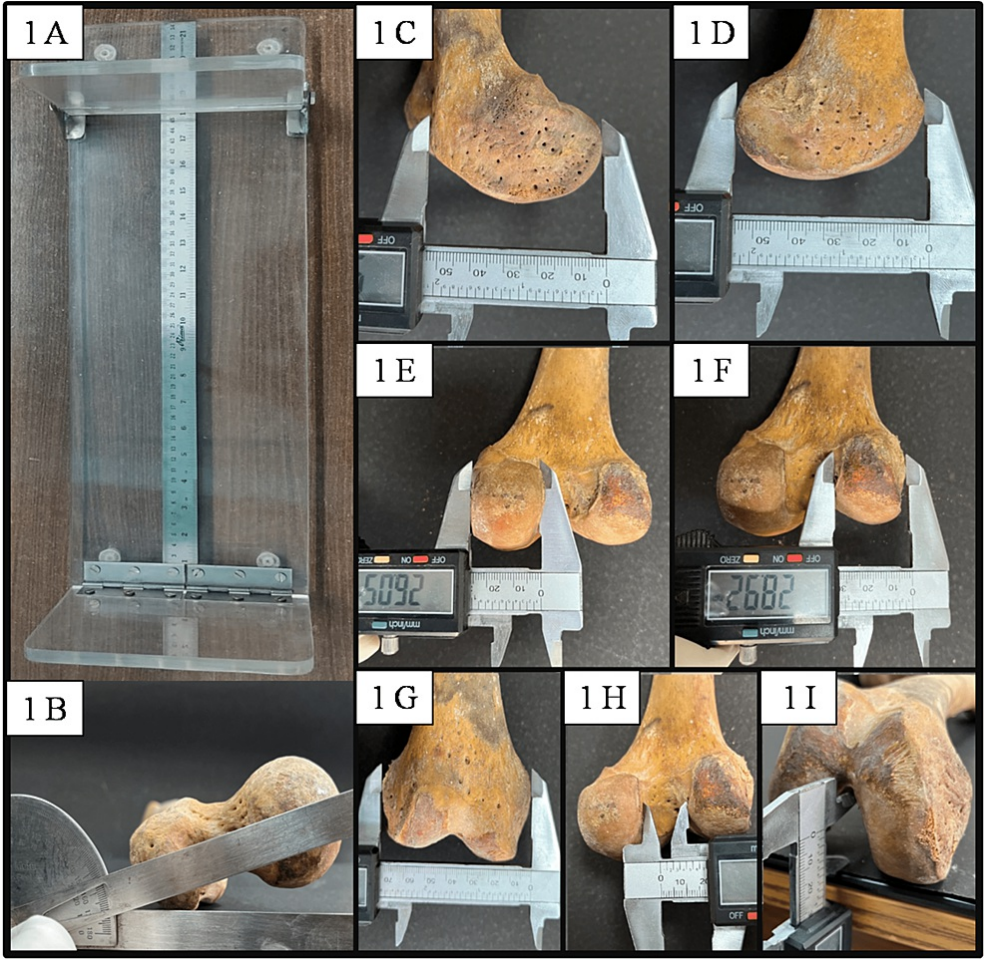
Measurements of femoral length by using osteometric board (1A), torsion angle by goniometer (1B) and different parameters of distal end of femur by using Vernier caliper (1C medial condyle length, 1D lateral condyle length, 1E medial condyle thickness, 1F lateral condyle thickness, 1G bicondylar width, 1H inter-condylar width and 1I inter-condylar depth)

Dry femora for the study were procured from the Department of Forensic Medicine, Institute of Medical Sciences, Banaras Hindu University, Varanasi, Uttar Pradesh, India. These femora were collected from corpses brought to the department for medicolegal autopsy; the details of the corpses were duly recorded. All the available femora were observed. Broken and damaged femora were excluded from the study. A total of 96 intact femora were included in this study, of which 51 were of the right side and 45 of the left side. Measurements were made using a digital Vernier caliper (accuracy 0.001 mm). The femoral length was measured using an osteometric table (Bio Enterprises, Aligarh, India) (Figure 1A). The torsion angle was measured with an analog goniometer (Figure 1B).

Medial condyle and lateral condyle length were measured as the maximum anteroposterior extension of respective condyles (Figure 1C, 1D). Medial condyle thickness and lateral condyle thickness were noted as the maximum side-to-side bony extension of the respective condyles (Figure 1E, 1F). Bicondylar width denotes the length between the lateral most bony part of the lateral condyle and the medial most bony point of the medial condyle (Figure 1G), while intercondylar width denotes the length between the medial most bony part of the lateral condyle and the lateral most bony part of the medial condyle (Figure 1H). The intercondylar depth denotes the distance between the most anterior point of the inferior border of the intercondylar notch and the horizontal plane on which the femur is kept (Figure 1I). Femur length is the maximum length measured from the tip of the head of the femur to the medial femoral condyle below. Torsion angle is defined as the angle between a line passing through the head, neck, and greater trochanter of the femur proximally and a line through femoral condyles distally.

Data obtained from measurements were noted on an Excel sheet (Microsoft, Redmond, WA, USA) and statistical analysis was done using SPSS version 25 (IBM Corp., Armonk, NY, USA). While observing the correlation between parameters, p-value <0.01 was considered highly significant and p-value <0.05 was considered statistically significant. Higher p-value indicates that there is no statistically significant correlation between the parameters.

## Results

In our study, the medial condyle length was observed to be 57.38±4.47mm with minimum condyle length being 46.12mm and a maximum length of 65.47mm, while the mean medial condyle thickness was 24.53±2.27mm. Mean lateral condyle length and thickness were calculated to be 58.49±4.3mm and 25.33±3.15mm respectively. The range of bicondylar width was from 54.58mm to 84.26mm with a mean of 71.96±6.73mm. Mean intercondylar width and intercondylar depth were found to be 21.86±2.71mm and 27.04±2.59mm respectively. The average femur length was 41.87±3.31cm, with the shortest being 23cm and the longest being 54.2cm. The minimum torsion angle was observed to be 3° while the maximum torsion angle was 46°. The average torsion angle was 20.19°±6.99° (Table 1).

**Table 1:** Descriptive Statistics MCL- medial condyle length; MCT- medial condyle thickness; LCL- lateral condyle length; LCT- lateral condyle thickness; BW- bicondylar width; ICW- intercondylar width; ICD- intercondylar depth; FL- femur length; TA- torsion angle.

	N	Minimum	Maximum	Mean		Std. Deviation
	Statistic	Statistic	Statistic	Statistic	Std. Error	Statistic
MCL	96	46.12	65.47	57.3778	.45707	4.47836
MCT	96	20.12	29.80	24.5335	.23149	2.26815
LCL	96	47.96	65.88	58.4883	.43685	4.28021
LCT	96	17.54	29.94	25.3250	.32193	3.15427
BW	96	54.58	84.26	71.9634	.68738	6.73488
ICW	96	15.86	29.12	21.8552	.27680	2.71209
ICD	96	21.55	33.46	27.0375	.26525	2.59891
FL	96	23.00	54.20	41.8679	.33805	3.31221
TA	96	3.00	46.00	20.1938	.71419	6.99759
Valid N (listwise)	96					

Medial condyle length was related significantly to medial condyle thickness, lateral condyle length, lateral condyle thickness, bicondylar width, intercondylar width, intercondylar depth and femur length. Medial condyle thickness was significantly related to medial condyle length, lateral condyle length, lateral condyle thickness, bicondylar width and intercondylar depth. Lateral condyle length was related significantly to the rest of the parameters observed. Lateral condyle thickness was related significantly to all the parameters except intercondylar width and torsion angle. Bicondylar width was related to all the parameters except torsion angle. Intercondylar width was significantly related to medial condyle length, medial condyle thickness, lateral condyle length, bicondylar width and femur length. Intercondylar depth was significantly related to all the parameters other than intercondylar width and torsion angle. Femur length was related significantly to all parameters except medial condyle thickness. Torsion angle was significantly related to lateral condyle length and femur length only (Table 2).

**Table 2:** Correlations between various parameters MCL- medial condyle length; MCT- medial condyle thickness; LCL- lateral condyle length; LCT- lateral condyle thickness; BW- bicondylar width; ICW- intercondylar width; ICD- intercondylar depth; FL- femur length; TA- torsion angle. **. Correlation is significant at the 0.01 level (2-tailed). *. Correlation is significant at the 0.05 level (2-tailed).

		MCL	MCT	LCL	LCT	BW	ICW	ICD	FL	TA
MCL	Pearson Correlation	1	.483^**^	.825^**^	.662^**^	.510^**^	.384^**^	.585^**^	.336^**^	-0.177
	Sig. (2-tailed)		0	0	0	0	0	0	0.001	0.085
	N	96	96	96	96	96	96	96	96	96
MCT	Pearson Correlation	.483^**^	1	.507^**^	.561^**^	.658^**^	-0.052	.545^**^	0.09	-0.023
	Sig. (2-tailed)	0		0	0	0	0.618	0	0.381	0.828
	N	96	96	96	96	96	96	96	96	96
LCL	Pearson Correlation	.825^**^	.507^**^	1	.648^**^	.628^**^	.432^**^	.694^**^	.543^**^	-.218^*^
	Sig. (2-tailed)	0	0		0	0	0	0	0	0.033
	N	96	96	96	96	96	96	96	96	96
LCT	Pearson Correlation	.662^**^	.561^**^	.648^**^	1	.451^**^	0.136	.550^**^	.211^*^	-0.074
	Sig. (2-tailed)	0	0	0		0	0.188	0	0.039	0.473
	N	96	96	96	96	96	96	96	96	96
BW	Pearson Correlation	.510^**^	.658^**^	.628^**^	.451^**^	1	.395^**^	.561^**^	.231^*^	-0.087
	Sig. (2-tailed)	0	0	0	0		0	0	0.023	0.401
	N	96	96	96	96	96	96	96	96	96
ICW	Pearson Correlation	.384^**^	-0.052	.432^**^	0.136	.395^**^	1	0.107	.274^**^	-0.049
	Sig. (2-tailed)	0	0.618	0	0.188	0		0.302	0.007	0.635
	N	96	96	96	96	96	96	96	96	96
ICD	Pearson Correlation	.585^**^	.545^**^	.694^**^	.550^**^	.561^**^	0.107	1	.322^**^	-0.178
	Sig. (2-tailed)	0	0	0	0	0	0.302		0.001	0.083
	N	96	96	96	96	96	96	96	96	96
FL	Pearson Correlation	.336^**^	0.09	.543^**^	.211^*^	.231^*^	.274^**^	.322^**^	1	-.241^*^
	Sig. (2-tailed)	0.001	0.381	0	0.039	0.023	0.007	0.001		0.018
	N	96	96	96	96	96	96	96	96	96
TA	Pearson Correlation	-0.177	-0.023	-.218^*^	-0.074	-0.087	-0.049	-0.178	-.241^*^	1
	Sig. (2-tailed)	0.085	0.828	0.033	0.473	0.401	0.635	0.083	0.018	
	N	96	96	96	96	96	96	96	96	96

## Discussion

The shape and parameters of the distal femur are important in determining knee mechanism. Various diseases like osteoarthritis and patellofemoral disorders are much influenced by distal femur parameters [7]. The parameters of the distal femur vary among race, sex, and region [2]. In our study, the mean medial condyle length was found to be 57.38±4.47mm, which is comparable to data found by Yazar et al. in Turkey (57±4.71mm), Khanal et al. in Nepal (57.4±4.1mm), Lakati et al. in Kenya (58.01mm) and Terzidis et al. in Greece (58.7±4.1mm) [2,8-10]. It was slightly lower than 63±5mm observed by Chandran et al. in south India and 61±4.4mm observed by Everhart et al. in Ohio [5,7].

The average lateral condyle length in the present study was observed to be 58.488±4.2mm which is lower than 60.94±4.5mm observed in Turkey by Yazar et al., 62.0±5.0mm observed in south India by Chandran et al., 62.3±4.3mm in Ohio by Everhart et al., and 61.2mm in Kenya by Lakati et al. [2,5,7,9]. Our data were comparable to that noted in Greece by Terzidis et al., 58.5±4mm [10]. Our data were higher than the Nepalese population by Khanal et al., 56±4.9mm [8].

The mean medial condyle thickness in this study is 24.53±2.26mm, comparable to that found by Yazar et al. in Turkey at 24.361±2.58mm and by Everhart et al. in Ohio at 24.2±3mm [2,7]. Average lateral condyle thickness was observed to be 25.32±3.15mm, slightly higher than that observed in Caucasians by Yazar et al. in Turkey, 23.61±2.18mm and by Everhart et al. in Ohio, 24.8±3mm [2,7].

Intercondylar width in the present study is 21.855±2.71mm, higher than that found in Turkey by Yazar et al. at 20.85±2.76mm and by Terzidis et al. in Greece at 20.5±2.2mm [2,10]. It was lower than the 23.04mm observed by Lakati et al. in Kenya [9].

Our average bicondylar width was noted to be 71.96±6.7mm, which correlates with the findings of Yazar et al., Chandran et al. and Prasad et al. at 71.13±5.24mm, 72±5mm and 71.4±4.2mm respectively [2,5,11]. It was higher than 69.7±6.7mm observed by Everhart et al. in Ohio and 68.44mm observed by Lakati et al. in Kenya [7,9]. It was quite less than 83.9±6.9mm noted by Terzidis et al. in Greece and 74.88±3.55mm noted in the male Malay population by Hussain et al. [10,12].

Intercondylar notch depth was calculated to be 27.04±2.6mm, higher than 26±2.34mm and 25.9±2mm found by Yazar et al. and by Terzidis et al. in Turkey and Greece respectively [2,10]. The mean femur length in our study was 41.87±3.31cm, comparable to that found by Yazar et al. in Turkey, 41.33±2.84cm [2]. It was less than 44.9±1.5cm, 46.12cm, 43.82±2.52cm and 43.02±2.3cm observed by Chandran et al. in south India, Lakati et al. in Kenya, Prasad et al. in Uttarakhand, India, and Lingamdenne et al. in Hyderabad, India respectively [5,9,11,13]. The mean torsion angle was found to be 20.19°±6.9°, higher than 12.3°±7.3° observed in south India by Prasad et al. and 15.1°±5.9° noted by Hudson et al. in the United States [14,15].

## Conclusions

Most of the parameters of the distal femur were significantly correlated with each other as well as with femur length. Lateral condyle length was the parameter that showed the maximum statistically significant correlation with the rest of the other parameters followed by medial condyle length and bicondylar width, which were significantly correlated with all parameters except torsion angle. Torsion angle was the parameter least related to other parameters, being significantly related to femur length and lateral condyle length only. We observed that femur length was most significantly related to lateral condyle length among all other parameters studied.

In the present study, we noted that values of medial condyle length and medial condyle thickness of the eastern Uttar Pradesh population are comparable to those of the Caucasian population. The data obtained on lateral condyle length in this study were less than that of different regions of the world and even that obtained from the southern part of India itself. The torsion angle value in our study was much higher than that of Caucasians and that of the southern part of India.

These findings further prove that the parameters of the distal femur are dependent on multiple factors and vary according to region. This should be considered by orthopedic surgeons and sports physicians during procedures as well as in graft designing. The data obtained here will also be helpful to forensic experts in routine practice.
